# Genetic diagnostic yields of 354 Chinese ASD children with rare mutations by a pipeline of genomic tests

**DOI:** 10.3389/fgene.2023.1108440

**Published:** 2023-03-23

**Authors:** Yue Zhang, Ying Li, Ruolan Guo, Wenjian Xu, Xuanshi Liu, Chunlin Zhao, Qi Guo, Wenshan Xu, Xin Ni, Chanjuan Hao, Yonghua Cui, Wei Li

**Affiliations:** ^1^ Beijing Key Laboratory for Genetics of Birth Defects, Beijing Pediatric Research Institute, MOE Key Laboratory of Major Diseases in Children, Beijing Children’s Hospital, Capital Medical University, National Center for Children’s Health, Beijing, China; ^2^ Department of Psychiatry, Beijing Children’s Hospital, Capital Medical University, National Center for Children’s Health, Beijing, China; ^3^ National Center for Children’s Health, Beijing, China

**Keywords:** autism spectrum disorder, whole-genome sequencing, RNA sequencing, copy number variation, single nucleotide variation

## Abstract

**Purpose:** To establish an effective genomic diagnosis pipeline for children with autism spectrum disorder (ASD) for its genetic etiology and intervention.

**Methods:** A cohort of 354 autism spectrum disorder patients were obtained from Beijing Children’s Hospital, Capital Medical University. Peripheral blood samples of the patients were collected for whole genome sequencing (WGS) and RNA sequencing (RNAseq). Sequencing data analyses were performed for mining the single nucleotide variation (SNV), copy number variation (CNV) and structural variation (SV). Sanger sequencing and quantitative PCR were used to verify the positive results.

**Results:** Among 354 patients, 9 cases with pathogenic/likely pathogenic copy number variation and 10 cases with pathogenic/likely pathogenic single nucleotide variations were detected, with a total positive rate of 5.3%. Among these 9 copy number variation cases, 5 were *de novo* and 4 were inherited. Among the 10 *de novo* single nucleotide variations, 7 were previously unreported. The pathological *de novo* mutations account for 4.2% in our cohort.

**Conclusion:** Rare mutations of copy number variations and single nucleotide variations account for a relatively small proportion of autism spectrum disorder children, which can be easily detected by a genomic testing pipeline of combined whole genome sequencing and RNA sequencing. This is important for early etiological diagnosis and precise management of autism spectrum disorder with rare mutations.

## Introduction

Autism spectrum disorder (ASD) is a group of neurodevelopmental disorder with onset at early childhood. According to the Diagnostic and Statistical Manual of Mental Disorders (Fifth Edition, DSM-V), autism, Asperger’s syndrome, childhood disintegrating disorder and unclassified pervasive developmental disorder are collectively referred to ASD (American Psychiatric Association, 2013). The two core symptoms of ASD are 1) impaired social communication and interaction, 2) repetitive and stereotyped behaviors, interests, and activities (Lobar, 2016). The incidence of ASD has been on the rise in recent years. According to the Centers for Disease Control and Prevention (CDC) reports, 1 in 54 children are diagnosed with ASD (Maenner et al., 2020), with a significantly higher proportion of male patients than that of female patients, and the ratio of male to female patients is about 4 : 1 (Rutherford et al., 2016; Loomes et al., 2017; Maenner et al., 2020). Except for the core symptoms, ASD is also accompanied by other comorbidities including but not limited to intellectual disability, epilepsy, facial deformities, neurological imaging abnormalities, movement disorders, attention deficit hyperactivity disorder (ADHD), aggressive behavior, cardiovascular abnormalities, gastrointestinal disorders, sleep disorders, convulsions, oppositional defiant disorder, anxieties, obsessions and compulsions and depression (Simonoff et al., 2008; Popow et al., 2021). Due to the wide spectrum of symptoms, missing diagnosis or misdiagnosis often occurs. Genetic diagnosis is now a routine method for precision diagnosis and intervention of ASD.

The clinical manifestations of ASD are diverse and complex, and its etiology is still largely unknown. ASD shows great heterogeneity in clinical symptoms and genetic alterations (Sandin et al., 2017). More attention has been paid to the genetic factors associated with ASD. Known genetic abnormalities are copy number variants (CNVs), *de novo* single nucleotide variants (SNVs), common genetic variants, mosaicism, non-coding and regulatory pathogenic variations, and inherited recessive variants (Zhang et al., 2021). More than 1,000 genes related to ASD have been described in the SFARI database (https://gene.sfari.org). The reported single gene mutation related to ASD accounts for about 5% (Gaugler et al., 2014), and CNV accounts for 8%–20% of ASD (Sebat et al., 2007). However, to date, at least 70% of the affected individuals have no known genetic etiology (Dias and Walsh, 2020).

The clinical implementation of trio-whole exome/genome sequencing (WES/WGS) has been a significant contribution to the discovery of *de novo* SNVs to autism risk (Neale et al., 2012; Sanders et al., 2012; Iossifov et al., 2014). As these variants usually link to a single gene, it is particularly important in emphasizing the underlying neurobiology of *de novo* SNVs associated with autism. CNVs refer to large deletions or duplications often involving in several genes. The association of phenotype with gene dosage exists, but the confirmation of relationship is often difficult. In 2007, comparative genomic hybridization was used to establish a significant association between *de novo* submicroscopic structural variation (SV) and autism (Sebat et al., 2007). From then on, more CNVs related to autism have been identified. In the genomics era, more genetic architectural changes including SNVs, CNVs and SVs are associated with ASD in different disease cohorts of different populations. Nevertheless, functional experiments are crucial for these validations to better understand the pathogenicity.

Enhanced bioinformatics analyses integrate evolutionary constraints to identify risk genes with a false discovery rate less than or equal to 0.1. In addition to utilizing probability of loss of function (pLI), missense badness, PolyPhen-2 constraint score, researchers are able to identify variants affecting gene functions by predicted impact (Lek et al., 2016). These analyses not only confirm enrichment of *de novo* loss-of-function mutations which affect highly constrained genes, but also identify pathogenic missense mutations. Currently, there are a variety of molecular testing platforms in diagnosing ASD. Trio-WES and CNV sequencing (CNVseq) or chromosomal microarray analysis (CMA) are commonly used first-tier techniques in molecular diagnosis of ASD. In the next-generation sequencing era, with the cost of sequencing declining, WGS is more widely applied to detect SNVs, CNVs and SVs simultaneously to uncover both coding and non-coding variants. RNA sequencing (RNAseq) can increase the diagnostic rate by assessing the gene expression changes. Thus, we here integrate both WGS and RNAseq for the genomic analysis of our ASD cohort to evaluate its efficacy and clinical application in a single-center level, in order to characterize the genetic etiology of the patients for both known ASD genes or new candidate ASD genes by focusing on *de novo* SNVs and CNVs.

## Materials and methods

### General patient information

**FIGURE 1:**
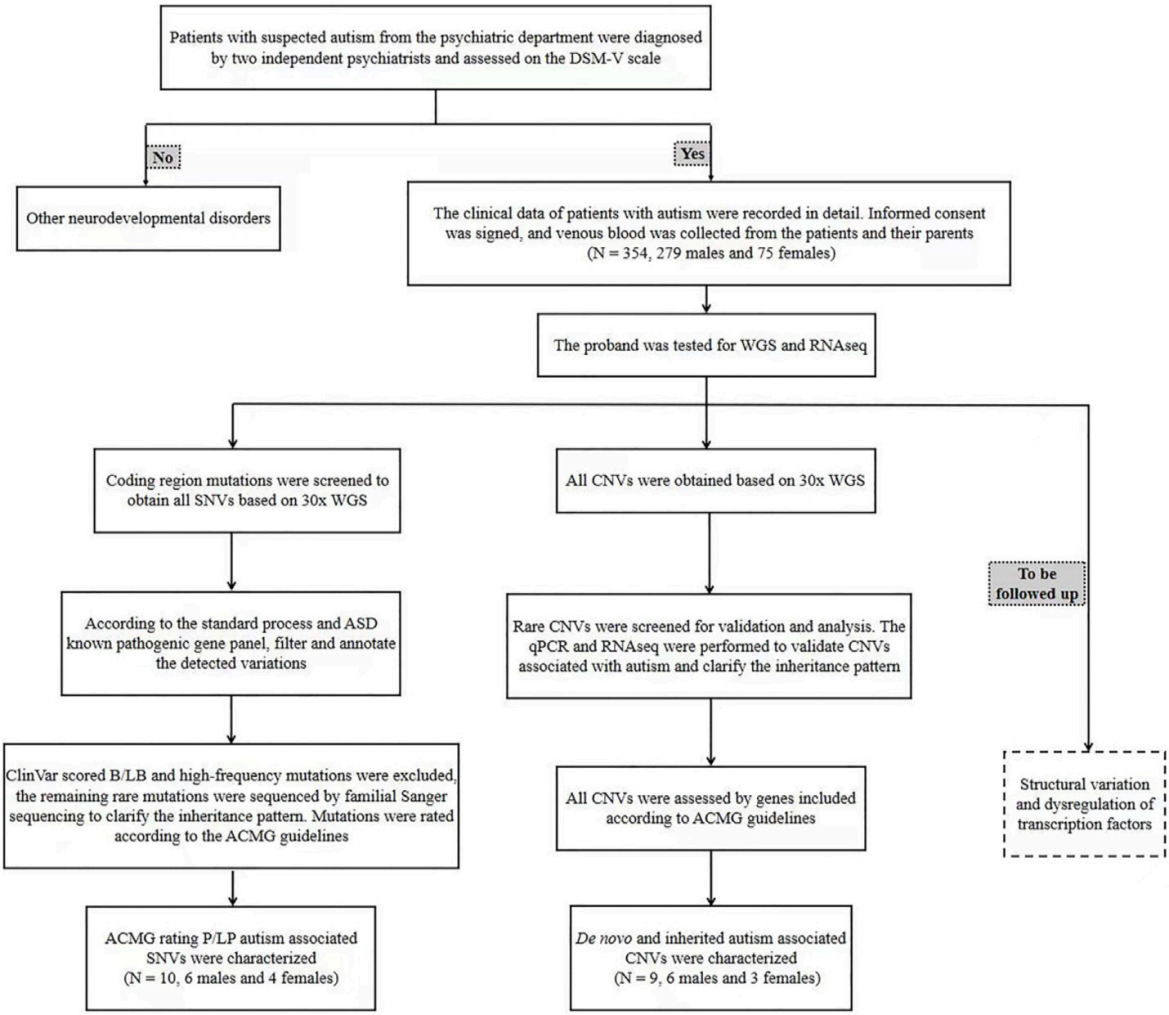
The clinical enrollment and diagnosis flowchart of ASD patients. qPCR: quantitative PCR; WGS: whole genome sequencing; SNV: single nucleotide variation; CNV: copy number variation; RNAseq: RNA sequencing.

The patients who met the diagnostic criteria of ASD by DSM-V were all from the Department of Psychiatry, Beijing Children’s Hospital. From July 2019 to May 2021, a total of 354 cases from 345 families were enrolled, including 9 families with two affected siblings. The ASD patients aged at 1–12 years, including 279 males and 75 females, with a male-to-female ratio of 3.72 : 1, very close to the ratio of male-to-female of about 4 : 1 (Maenner et al., 2020). The clinical data, imaging reports (if any) and ASD assessment scales of the patients were collected, including the Autism Behavior Checklist (ABC), Clancy Autism Behavior Scale (CABS), Childhood Autism Rating Scale (CARS) and DSM-V Diagnostic Scale (Supplementary Material). The head circumference and eye distance of the children were measured, and the front and side images were captured. The enrollment and diagnosis process are summarized in Figure 1. 5 mL of venous blood from the patient and 2 mL of venous blood from each parent were collected, and genomic DNA and total RNA were extracted. All parents have signed their own written informed consents as well as in representative of their children’s guardians. This study has been approved by the Ethics Committee of Beijing Children’s Hospital.

### Whole genome sequencing (WGS)

DNA of all collected samples was extracted and purified using DNA Blood Mini Kit (Qiagen, Hilden, Germany). DNA concentration was measured by Qubit DNA Assay Kit in Qubit 2.0 Flurometer (Life Technologies, CA, USA).

#### Library preparation

A total amount of 1 µg DNA sample from each proband was used as the input material for DNA library preparation. Sequencing library was generated using CleanNGS DNA Kit (CleanNA, Waddinxveen, Netherlands) following the manufacturer’s recommendations and index codes were added to each sample. Briefly, genomic DNA sample was enzymatically digested to fragments of about 350 bp. Then DNA fragments were end-polished, A-tailed, and ligated with the full-length adapter for sequencing, followed by further PCR amplification. After PCR products were purified by AMPure XP System (Beckman Coulter, CA, USA), libraries were analyzed for size distribution by Agilent 2100 Bioanalyzer (Agilent Technologies, CA, USA) and quantified by real-time PCR.

#### Clustering and sequencing

The clustering of the index-coded samples was performed on a cBot Cluster Generation System using Novaseq5000/6000 S4 Reagent Kit (Illumina, San Diego, CA, USA) according to the manufacturer’s instructions. After cluster generation, the DNA libraries were sequenced on Illumina NovaSeq 6000 platform and 150 bp paired-end reads were generated.

#### Quality control

Raw data (raw reads) of fastq format were firstly processed through primary quality control. In this step, clean data (clean reads) were obtained by removing read pairs that contain more than three N or the proportion of base with quality value below 5 is more than 20%, in any end, or adapter sequence was founded. All the downstream analyses were based on the clean data with high quality.

#### Reads mapping to reference sequences

Clean reads were compared with reference human genome (UCSC hg19) by using BWA software (http://bio-bwa.sourceforge.net), and the results were converted into bam format and sorted by Samtools software (https://github.com/samtools/samtools/releases). Finally, basic information statistics and map comparison statistics were conducted. The average depth of WGS is about 30×.

### RNA sequencing (RNAseq)

Total RNA of all collected samples was extracted and purified from the fresh venous whole blood using PAXgene Blood RNA Kit (Qiagen). Total RNA concentration was measured by Qubit RNA Assay Kit in Qubit 2.0 Flurometer (Life Technologies).

#### RNA quality check

The purity of the sample was determined by NanoPhotometer (Implen, CA, USA), the concentration and integrity of RNA samples were detected by Agilent 2100 RNA Nano 6000 Assay Kit.

#### Library preparation

A total amount of 1–3 μg RNA per sample from each proband was used as the input material for RNA sample preparation. Sequencing libraries were generated using VAHTS Universal V6 RNA-seq Library Prep Kit (Illumina, NR604-01/02) following the manufacturer’s recommendations and index codes were added to attribute sequences to each sample. Briefly, mRNA was purified from total RNA using poly-T oligo-attached magnetic beads. Then we added fragmentation buffer to break the mRNA into short fragments. First strand cDNA was synthesized using random hexamer primer and RNase H. Second strand cDNA synthesis was subsequently performed using buffer, dNTPs, DNA polymerase I and RNase H. The double stranded cDNA was purified by AMPure XP beads (Beckman Coulter). The purified double stranded cDNA was repaired at the end, added a tail and connected to the sequencing connector, then the fragment size was selected, and the final cDNA library was constructed by PCR enrichment.

#### Library check

RNA concentration of library was measured using Qubit RNA Assay Kit in Qubit 3.0 (Life Technologies) for quantification and then diluted to 1 ng/μL. Insert size was assessed using the Agilent Bioanalyzer 2100 system. After the insert size met the requirement, the CFX 96 fluorescence quantitative PCR instrument (Bio-Rad, CA, USA) was used to quantify the library effective concentration (library effective concentration >10 nm) using Bio-Rad iQ SYBR GRN Kit.

#### Library clustering and sequencing

1) HiSeq x ten platform (Illumina). The clustering of the index-coded samples was performed on a cBot cluster generation system using HiSeq PE Cluster Kit v4-cBot-HS according to the manufacturer’s instructions. After cluster generation, the libraries were sequenced on an Illumina platform and 150 bp paired-end reads were generated. 2) Novaseq 6000 S4 platform (Illumina). The cluster generation and sequencing were performed on Novaseq 6000 S4 platform, using NovaSeq 6000 S4 Reagent Kit V1.5.

### Sanger sequencing

Amplification primers were designed for the gene variants, and PCR amplification and sequencing verification were performed on the gene products in the family of the indicated patient. ABI 3730XL sequencer (Applied Biosystem, CA, USA) was used for Sanger sequencing.

### Quantitative PCR (qPCR)

The CNVs were validated by quantitative PCR (qPCR). qPCR validation was performed using the Roche LightCycler 480 Ⅱ System (Roche, Basel, Switzerland). One pair of primers was selected from the middle of each CNV. The samples from the family of the indicated patient were analyzed in triplicate in a 10 μL reaction mixture (200 nM each primer, LightCycler 480 SYBR Green Master Mix (2X) and 5 or 10 ng of genomic DNA). The values were evaluated using LightCycler 480 Software (Applied Biosystems). Data analysis was performed using the qBase method (Hellemans et al., 2007). *GAPDH* was used as the reference gene for the minimal coefficient of variation. Data were normalized by setting a normal control to a value of 1.

### Bioinformatic analyses of sequencing data and variants

Verita TreKKer (v1.9.3, Berry Genomics, Beijing, China) was used to identify SNP/InDels. CNVnator(v1.2.2) (Abyzov et al., 2011) and BIC-Seq (v0.7.2) (Xi et al., 2011) were used for CNV detection, while Manta (Chen et al., 2016) was used for SV discovery. EnliVen (v1.9.3, Berry Genomics) was performed to annotate SNP/InDels/CNV/SV. EnliVen and ANNOVAR (Wang et al., 2010) were executed for VCF (variant call format) files. dbSNP (http://www.ncbi.nlm.nih.gov/snp), 1,000 Genomics Project (http://browser.1000genomes.org) and other related existing databases were applied to annotate the variants. By focusing on exonic variants, gene transcript annotation databases, such as Consensus CDS protein set (http://www.ncbi.nlm.nih.gov/CCDS), RefSeq Gene (http://www.ncbi.nlm.nih.gov/RefSeq), Ensembl (https://www.ensembl.org) and UCSC (http://genome.ucsc.edu), were used to determine amino acid changes. Variants were analyzed for pathogenicity according to the American College of Medical Genetics and Genomics (ACMG) grading criteria (Richards et al., 2015).

The clean reads were aligned on genome hg19 along with annotated genes in the Ensembl website using STAR v2.7 aligner (Dobin et al., 2013). Gene expression level was quantified using DROP pipeline with the default parameters (Yépez et al., 2021). Differential gene expression was used to validate the effect of candidate SNVs and CNVs if the gene is stably expressed in blood. Candidate CNV regions cover up to hundreds of in-CNV genes. The average expression level of in-CNV genes were compared to that of equal number of randomly selected out-CNV genes outside the CNV region on the same chromosome. The difference between in-CNV and out-CNV genes was quantified by Z-score and log2-fold change. The duplication/loss CNVs were expected to show greater/smaller Z-score and log2-fold change, respectively.

## Results

### Intellectual disability and language delay are the two most common features of ASD

**FIGURE 2:**
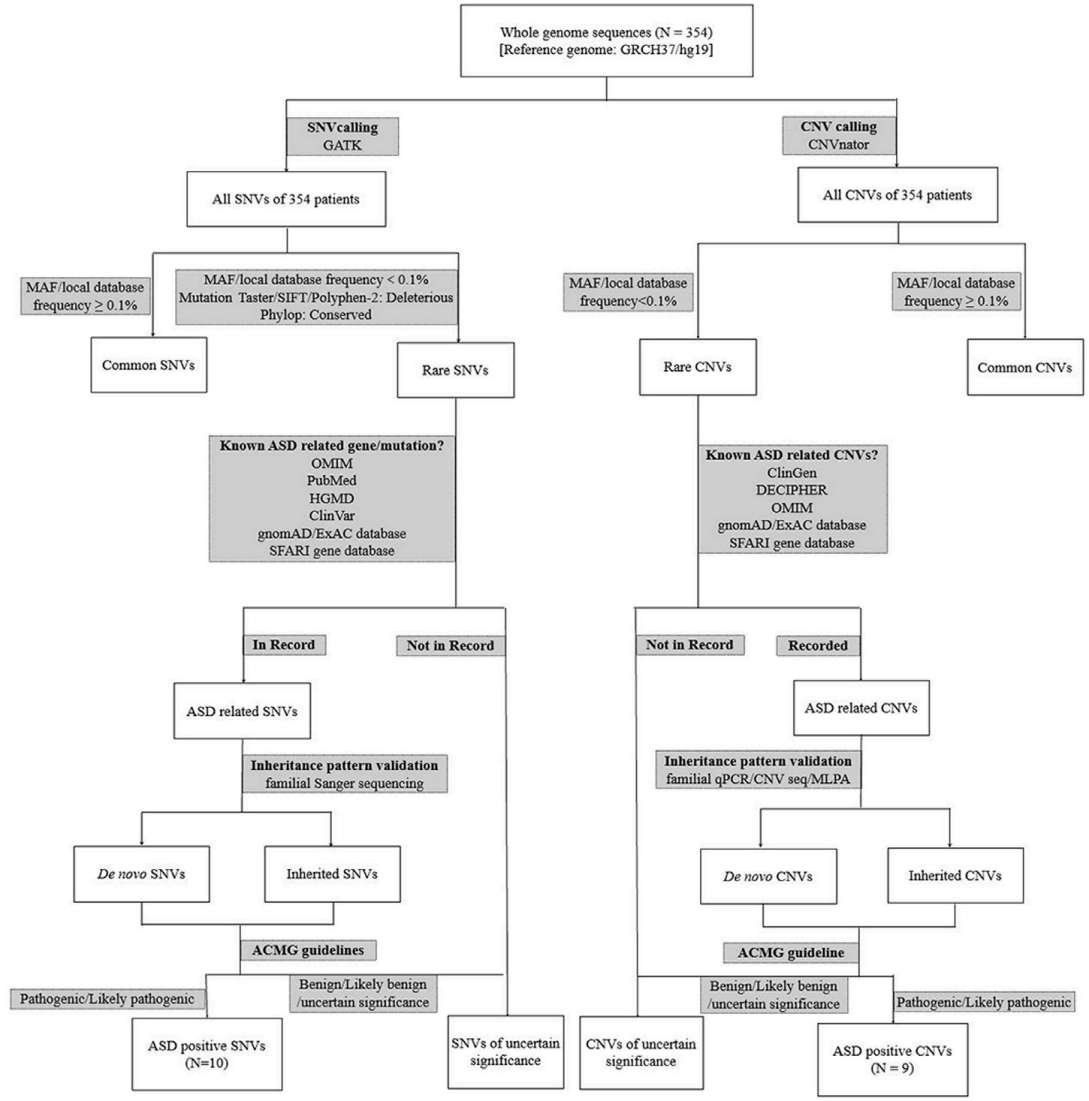
The detailed genomics diagnosis pipeline of ASD patients. qPCR: quantitative PCR; WGS: whole genome sequencing; SNV: single nucleotide variation; CNV: copy number variation; CNV seq: CNV sequencing.

Among 354 patients enrolled, 19 were molecular positive, including 12 males and 7 females, aged from 2 to 6 years. The diagnosis pipeline is summarized in Figure 2. The male-to-female ratio is less than about 4:1 as these positive cases were mostly having *de novo* mutations. The specific clinical features of 19 patients with positive diagnosis are shown in Table 1. Except for the ASD0330 patient who lost follow-up, 17 of the remaining 18 patients had clinical symptoms of intellectual disability and language delay which are less common in those molecularly unknown ASD patients, suggesting that the co-occurrence of these two symptoms could be suggestive of inborn errors in genetic alterations in ASD patients.

**TABLE 1:** Clinical features of 19 diagnosed ASD patients.

ID	Sex	Age (year)		Featured symptoms
ASD0018	male	3		Intellectual disability, language development delay
ASD0027	male	3		Severe intellectual disability, language retardation, wide dental space, abnormal posture
ASD0046	female	4		Intellectual disability, language retardation, attention deficit, hyperactivity, feeding difficulties
ASD0060	male	2		Intellectual disability, language development delay, sleep disorders, forehead protrusion
ASD0061	male	5		Intellectual disability, language development delay, global developmental delay
ASD0063	female	3		Intellectual disability, language retardation, movement disorders, epilepsy
ASD0134	male	3		Intellectual disability, language retardation, hyperactivity, strabismus, unsteady walking
ASD0144	male	2		Intellectual disability, language development delay, learning disabilities, cognitive disorders, attention deficit, feeding difficulties
ASD0148	female	6		Intellectual disability, language retardation, motor retardation, increased pain tolerance, spherical nasal tip
ASD0162	male	2		Intellectual disability, language deficits, learning disabilities, large head, scoliosis, multiple milky coffee spots
ASD0203	female	6		Attention deficit, hyperactivity, global developmental delay
ASD0214	female	4		Intellectual disability, language deficits, global developmental delay, cognitive disorders, feeding difficulties
ASD0219	female	3		Mild intellectual disability, language development delay, cognitive disorders, hyperactivity, gross/fine motor retardation
ASD0221	male	3		Intellectual disability, language absence, motor retardation, instability of gait
ASD0222	female	3		Mild intellectual disability, language development delay, motor retardation
ASD0294	male	5		Intellectual disability, language development delay, global development delay
ASD0326	male	4		Mild intellectual disability, language development delay, global development delay
ASD0330	male	3		lost of follow-up
ASD0343	male	5		Intellectual disability, language development delay, cognitive disorders, learning disabilities, hyperactivity

### CNVs detected in this ASD cohort

We applied low-depth WGS CNVseq-algorithm to analyze genomic alterations in 354 ASD patients. The distribution and burden analysis of CNV calls were calculated and summarized in Supplementary Figure S1. Among these CNVs, we found 9 cases were positive for pathological CNVs (Table 2). The resolution of CNVs reached 100 kb (Lord et al., 2018). To verify the CNV results, qPCR was performed on the positive cases, which are all in agreement (Supplementary Figure S2). Among these CNVs, 6 were duplicated CNVs, other 3 were deletional CNVs. Some of these deletions/duplications are known syndromes. Among these 9 CNV cases, 5 were *de novo* and 4 were inherited.

**TABLE 2:** CNV changes in 9 ASD patients.

ID	Sex	Chromosome region	CNV site	Type of change	Size (Mb)	Origin	Clinical diagnosis	Affected parents’ featured symptoms (if any)
ASD0018	male	22q11.2	chr22: 18910690-21463171	duplication	2.7	*de novo*	22q11.2 duplication (proximal, A-D) syndrome	-
ASD0027	male	7q11.23	chr7: 72688896-74173668	duplication	1.6	*de novo*	Williams-Beuren syndrome critical region	-
ASD0060	male	2p16.3	chr2: 51046035-51696622	deletion	0.7	paternal	2p16.3 deletion syndrome	Normal
ASD0144	male	16p13.11	chr16: 14901699-16492313	duplication	1.49	maternal	16p13.11 recurrent microduplication syndrome	Normal
ASD0203	female	15q11q13	chr15: 23596697-28012138	duplication	4.4	maternal	15q11q13 recurrent (PWS/AS) region (BP2-BP3, Class 2)	Normal
ASD0214	female	3q26	chr3: 176244949-178219435	deletion	2	*de novo*	3q26 deletion include *TBL1XR1* gene (ASD related gene)	-
ASD0222	female	3q29	chr3: 195740002-197962430	deletion	1.6	*de novo*	3q29 recurrent region (includes *DLG1* gene)	-
ASD0330	male	15q11q13	chr15: 23597027-28729564	duplication	4.6	*de novo*	15q11q13 recurrent (PWS/AS) region (BP2-BP3, Class 2)	-
ASD0343	male	1q21.1	chr1: 143691670-148830060	duplication	5	maternal	1q21.1 recurrent region (BP3-BP4, distal) (includes *GJA5* gene)	Congenital heart disease

#### Duplications

ASD0018 carried a *de novo* duplication of the 22q11.2 proximal (A-D) region, which is associated with a highly variable clinical phenotype, ranging from apparently normal to a broad range of clinical features, including non-specific phenotypes (intellectual disability, learning disability, developmental delays, autism, psychiatric disorder growth delays, hypotonia) as well as phenotypes that overlap clinical findings of DiGeorge syndrome (DGS; OMIM #188400) or velocardiofacial syndrome (VCFS; OMIM #192,430) (ClinGen, 2018). 22q11.2 duplications are frequently inherited and incomplete penetrance has been demonstrated. The 22q11.2 duplication is often found in an apparently normal parent of a proband (Firth, 1993).

ASD0027 carried a *de novo* duplication of the 7q11.23 recurrent region (Williams-Beuren syndrome, OMIM #609757, including the *ELN* gene). Clinical findings in this syndrome may include speech delay, autistic features, motor delay, seizures, hypotonia, brain anomalies, joint laxity, and craniofacial abnormalities. Williams-Beuren region duplication syndrome is considered to be highly penetrant with patients showing variable expressivity. Both inherited and *de novo* cases of 7q11.23 duplications have been reported in the literature. The population frequency of the chromosome 7q11.23 duplication syndrome was estimated to be 1 in 13,000 to 20,000 (Van der Aa et al., 2009; Levy et al., 2011). This patient presented wide dental space, which has not been reported.

ASD0144 carried a duplication of the 16p13.11 recurrent region (BP2-BP3) (including the *MYH11* gene) inherited from his phenotypically normal father. Duplication of the 16p13.11 region has been associated with a variable clinical presentation including developmental delay, intellectual disability/learning difficulties, behavioral abnormalities (including ASD and ADHD), hypotonia, congenital heart disease (such as tetralogy of Fallot), and variable dysmorphic features. The majority of patients with this duplication are inherited, mostly from an apparently unaffected parent or a parent with abnormal phenotypes similar to the child’s phenotype but not that much severe (Khattabi et al., 2020). Penetrance for any clinical phenotype associated with this duplication was estimated to be 7% (Kendall et al., 2019).

ASD0203 and ASD0330 both carried a duplication of 15q11q13 recurrent region (PWS/AS, BP2-BP3, Class 2), which is associated with chromosome 15q11-q13 duplication syndrome (OMIM #608636). Common clinical symptoms of this syndrome are autism, intellectual disability, ataxia, epilepsy, developmental delay and mental and behavioral problems (Bundey et al., 1994; Burnside et al., 2011). Most of the reported cases were familial inherited cases, and few were *de novo* mutations (Christian et al., 2008). The syndrome showed incomplete penetrance, and the clinical manifestations of different patients were highly heterogeneous. ASD0203 inherited the duplication from her phenotypic normal mother, while ASD0330 carried a *de novo* duplication.

ASD0343 carried a duplication of the 1q21.1 recurrent region (BP3-BP4, distal, including the *GJA5* gene) inherited from his mother, who had congenital heart disease. Duplication of the 1q21.1 region has been associated with chromosome 1q21.1 duplication syndrome (OMIM #612475). The clinical phenotypes of the syndrome include varying degrees of intellectual impairment, macrocephaly, hypotonia, congenital heart disease (such as tetralogy of Fallot), and malformation features. Mental and behavioral disorders include ASD, ADHD and others. Most of the reported cases were inherited from unaffected parents with normal or abnormal phenotypes similar to the child but not severe, and the clinical phenotypes of patients are highly variable (Brunetti-Pierri et al., 2008; Bernier et al., 2016).

#### Deletions

ASD0060 carried a deletion at 2p16.3 involving exons of a haploinsufficiency gene *NRXN1* inherited from his phenotypic normal father, which is associated with susceptibility to autism, schizophrenia (SCZD17), developmental delay, intellectual disability, and dysmorphic features. The phenotypes are highly variable and show incomplete penetrance (Schaaf et al., 2012; Dabell et al., 2013). Sleeping disorder of ASD0060 has not been reported in previous studies.

ASD0214 carried a *de novo* deletion covering a haploinsufficiency gene *TBL1XR1*, Heterozygous mutation or deletion of this gene is associated with autosomal dominant inherited intellectual disability type 41. The common clinical symptoms of patients include autism, intellectual disability, developmental delay, spasticity and facial deformations. The clinical manifestations of different patients are highly heterogeneous (O'Roak et al., 2012a; O'Roak et al., 2012b; Saitsu et al., 2014).

ASD0222 carried a *de novo* deletion of the 3q29 recurrent region (including the *DLG1* gene). Deletion of this region is associated with chromosome 3q29 deletion syndrome (OMIM #609425). Clinical findings in this syndrome are mild to moderate developmental delay, intellectual disability, ASD, speech delay, walking delay, microcephaly and mild dysmorphic features. Most 3q29 deletions are *de novo* mutations, and a small number of cases are inherited from parents (Ballif et al., 2008; Quintero-Rivera et al., 2010; Città et al., 2013).

### SNVs detected in this ASD cohort

We used the 30× WGS data for analyzing SNVs. Among 354 children with ASD, 10 were found with *de novo* deleterious SNVs (non-sense splicing or frameshift mutation, Table 3). The mutation sites and parental origins were verified by Sanger sequencing (Supplementary Figure S3). Among these mutational alleles, 7 were previously unreported alleles (PUAs).

**TABLE 3:** SNV changes of 10 ASD patients.

ID	Sex	Gene	Nucleotide change	Amino acid change	Type of mutation	Origin	Clinical diagnosis
ASD0046	female	*ASH1L*	c.8595delT	p.Gln2866fs	Frameshift	*de novo*	Intellectual developmental disorder, autosomal dominant 52
ASD0061	male	*EP300*	c.4242T>G	p.Tyr1414*	Non-sense	*de novo*	Menke-Hennekam syndrome 2
ASD0063	female	*SCN2A*	c.4550_4551del	p.Ala1517fs	Frameshift	*de novo*	Developmental and epileptic encephalopathy 11
ASD0134	male	*ADNP*	c.2156_2157insA	p.Tyr719*	Non-sense	*de novo*	Helsmoortel-van der Aa syndrome
ASD0148	female	*SHANK*3	c.4728_4740del	p.Leu1577fs	Frameshift	*de novo*	Phelan-McDermid syndrome
ASD0162	male	*NF1*	c.7395-2A>T	-	Splicing	*de novo*	Neurofibromatosis, type 1
ASD0219	female	*PRKD1*	c.41_71del	p.Leu14fs	Frameshift	*de novo*	Congenital heart defects and ectodermal dysplasia
ASD0221	male	*SCN2A*	c.605 + 1G>A	-	Splicing	*de novo*	Episodic ataxia type 9
ASD0294	male	*PTEN*	c.546dupA	p.Leu182fs	Frameshift	*de novo*	Macrocephaly/autism syndrome
ASD0326	male	*SCN2A*	c.1570C>T	p.Arg524*	Non-sense	*de novo*	Episodic ataxia type 9

ASD0063, ASD0221 and ASD0326 all carried *de novo* mutations of the *SCN2A* gene, which encodes the α-subunit family of voltage-gated sodium channel that is responsible for the action potentials in neurons and muscles. Mutations in *SCN2A* have been linked to epilepsy and ASD (Sanders et al., 2012; Tavassoli et al., 2014). The c. 4550_4551del of the *SCN2A* gene in ASD0063 was a PUA, while the c.605 + 1G>A mutation in ASD0221 and the c.1570C>T mutation in ASD0326 have been previously reported. ASD0063 had developmental and epileptic encephalopathy, seizures, benign familial infantile. ASD0221 and ASD0326 had no seizures, which is consistent with the clinical symptoms of patients with the same mutation reported previously (Kothur et al., 2018; van der Werf et al., 2020).

ASD0046 carried a PUA c. 8595delT of the *ASH1L* gene. The histone methyltransferase encoded by this gene is involved in chromatin epigenetic modifications and is associated with the transcriptional regulation of developmentally important genes. Mutations in the *ASH1L* gene can lead to intellectual developmental disorder, autosomal dominant 52. About 60% of the patients have ASD (Stessman et al., 2017).

ASD0061 carried a PUA c.4242T>G of the *EP300* gene, which encodes p300, a histone acetyltransferase that regulates transcription *via* chromatin remodeling and is important in the processes of cell proliferation and differentiation. Mutations in the *EP300* gene can lead to Menke-Hennekam syndrome-2 (MKHK2), characterized by variable impairment of intellectual development and facial dysmorphisms. Feeding difficulties, autistic behavior, recurrent upper airway infections, and hearing impairment are also frequently seen (Hamilton et al., 2016; Menke et al., 2018).

ASD0134 carried a *de novo* c.2156dupA mutation of the *ADNP* gene, which encodes a zinc finger protein with a homeodomain that has the transcription factor activity and is essential for brain formation. Mutations of the *ADNP* gene can lead to Helsmoortel-van der Aa syndrome, which includes intellectual disability, developmental delay, ASD and facial dysmorphic features (Helsmoortel et al., 2014; Breen et al., 2020). The incidence of this gene mutation in ASD population is 0.17% (Helsmoortel et al., 2014). The c.2156dupA of *ADNP* gene has been reported. Patients with this mutation had neurodevelopmental disorder, ADNP-related multiple congenital anomalies, intellectual disability, and ASD (DDD Study, 2015; Chérot et al., 2018).

ASD0148 carried a PUA c.4728_4740del of the *SHANK3* gene. The scaffold protein encoded by this gene is enriched in the postsynaptic compact of excitatory synapses and is associated with epithelial tubule development and excitatory synaptic transmission in the renal and enteric nervous systems. Mutations in the *SHANK3* gene can lead to Phelan McDermid syndrome (Prasad et al., 2000). Phelan McDermid syndrome is a developmental disorder with a variety of abnormal clinical manifestations, including neonatal hypotonia, global developmental delay, normal to accelerated growth, severe language delay to language loss, autistic behavior, and mild dysmorphic features (Prasad et al., 2000; Durand et al., 2007). Some studies have shown that mutations in the *SHANK3* gene are found in about 0.75% of ASD patients (Moessner et al., 2007). ASD0148 had increased pain tolerance, which has not been previously reported.

ASD0162 carried a PUA c.7395-2A>T of the *NF1* gene. This gene encodes neurofibrin, which is mainly expressed in neurons, Schwann cells, oligodendrocytes and leukocytes. Mutations in the *NF1* gene can lead to neurofibroma type I, and about 10%–40% of patients with neurofibroma type I have been reported to have ASD (Walsh et al., 2013; Eijk et al., 2018).

ASD0219 carried a PUA c.41_71del of the *PRKD1* gene. The protein encoded by *PRKD1* is a serine/threonine kinase that regulates a variety of cellular functions, including membrane receptor signaling, transport at the Golgi, protection from oxidative stress at the mitochondria, gene transcription, and regulation of cell shape, motility, and adhesion. Mutations in the *PRKD1* gene can lead to congenital heart defects and ectodermal dysplasia (Sifrim et al., 2016). Case-control studies have shown that *de novo* mutations in the *PRKD1* gene occur more frequently in patients with autism than in the general population (Ellaway et al., 2013; Iossifov et al., 2014; Rubeis et al., 2014; DDD Study, 2015).

ASD0294 carried a PUA c.546dupA of the *PTEN* gene. This gene encodes a ubiquitously expressed tumor suppressor dual-specificity phosphatase that antagonizes the PI3K signaling pathway through its lipid phosphatase activity and negatively regulates the MAPK pathway through its protein phosphatase activity. Mutations in the *PTEN* gene lead to macrocephaly/autism syndrome, characterized by increased head circumference, abnormal facial features, and delayed psychomotor development resulting in autistic behavior or intellectual disability (Herman et al., 2007; Page et al., 2009). Some patients may have a primary immunodeficiency disorder with recurrent infections associated with variably abnormal T- and B-cell function (Tsujita et al., 2016).

### RNAseq validation of positive cases in this ASD cohort

RNAseq is often used for candidate disease gene discovery. Hear we use RNAseq to evaluate the expressional changes in the blood samples of the ASD patients with deleterious mutations.

#### RNA expression level of detected positive CNVs

For each positive CNV, we first calculate background expression level of all genes located on the same chromosome of the patient. Next, we randomly selected three set of out-CNV genes on the chromosome where the CNV was located as negative controls of the patient. Then we calculated the average expression level of in-CNV and out-CNV genes. Finally, we calculated the value of z-score and log2-fold change of the in-CNV and out-CNV genes. Except that the genes affected in ASD0060 were not expressed in the peripheral blood, the z-score values and log2-fold changes of all other positive CNV cases were consistent with our genomic analysis results (duplication or deletion) (Table 4).

**TABLE 4:** RNAseq results of positive CNVs detected in this ASD cohort.

ID	Chromosome region	Type of change	gene_in_cnv	RNA_in_cnv	zScore_ chr	zScore_ cnv	zScore_ random1	zScore_ random2	zScore_ random3	log2fc_ chr	log2fc_ cnv	log2fc_ random1	log2fc_ random2	log2fc_ random3
ASD0018	22q11.2	Duplication	92	38	0.04	0.38	−0.1	0.145	0.095	−0.01	0.05	−0.015	0.015	0
ASD0027	7q11.23	Duplication	35	16	0.095	3.745	0.345	0.245	−0.155	0	0.48	0.03	0.02	−0.01
ASD0060	2p16.3	deletion	1	0	-	-	-	-	-	-	-	-	-	-
ASD0144	16p13.11	duplication	42	17	−0.06	2.3	−0.29	−0.03	0.5	−0.015	0.38	−0.06	−0.01	0.06
ASD0203	15q11q13	duplication	122	3	0.06	2.56	0.8	−0.09	−0.85	0	0.38	0.1	−0.03	−0.12
ASD0214	3q26	deletion	15	1	0.03	−4.86	−1.26	1.74	0.69	−0.01	−0.41	−0.1	1.12	0.19
ASD0222	3q29	deletion	59	22	0.05	−5.025	0.205	−0.23	0.01	0	−0.775	0.01	−0.04	−0.005
ASD0330	15q11q13	duplication	130	5	−0.01	1.41	0.31	−0.23	0.46	−0.01	0.32	−0.06	−0.04	0.09
ASD0343	1q21.1	duplication	99	18	0.07	2.075	0.205	0.12	−0.065	0	0.385	0.02	0.005	−0.015

Gene_in_cnv: gene numbers involved in CNV; RNA_in_cnv: gene numbers expressed in blood of the CNV; zScore_chr: Z-score value of the whole chromosome of the CNV; zScore_cnv: Z-score value of the CNV; zScore_random1/2/3: Z-score value of randomly selected non-mutated locations on the chromosome where the CNV is located; log2fc_chr: log2-fold change value of the whole chromosome of the CNV; log2fc_cnv: log2-fold change value of the CNV; log2fc_random1/2/3: log2-fold change value of randomly selected non-mutated locations on the chromosome where the CNV is located.

#### RNA expression level of detected positive SNVs

We calculated the mutant gene expression level for SNV positive cases and used the average expression level of this gene in all other patients as the control. Several cases (the *SCN2A* gene of ASD0063, ASD0221 and ASD0326, the *SHANK3* gene of ASD0148 and the *PRKD1* gene of ASD0219) were excluded from this validation analysis because these genes were not expressed in peripheral blood. Indeed, the expression of mutant genes in the other five SNV-positive cases decreased compared with the average value of RNA expression level of 354 patients (Table 5).

**TABLE 5:** RNAseq results of positive SNVs detected in this ASD cohort.

ID	Gene	Nucleotide change	Type of mutation	Ensemble ID	Express in blood	Patient RNA expression level	Mean RNA expression level
ASD0046	*ASH1L*	c.8595delT	Frameshift	ENSG00000116539	yes	1,196.02	1,206.834
ASD0061	*EP300*	c.4242T>G	Non-sense	ENSG00000100393	yes	2520.55	2718.56
ASD0063	*SCN2A*	c.4550_4551del	Frameshift	ENSG00000136531	no	-	-
ASD0134	*ADNP*	c.2156_2157insA	Non-sense	ENSG00000101126	yes	1,663.37	1791.54
ASD0148	*SHANK*3	c.4728_4740del	Frameshift	ENSG00000251322	no	-	-
ASD0162	*NF1*	c.7395-2A>T	Splicing	ENSG00000196712	yes	534.96	615.19
ASD0219	*PRKD1*	c.41_71del	Frameshift	ENSG00000184304	no	-	-
ASD0221	*SCN2A*	c.605 + 1G>A	Splicing	ENSG00000136531	no	-	-
ASD0294	*PTEN*	c.546dupA	Frameshift	ENSG00000171862	yes	3095.66	3948.45
ASD0326	*SCN2A*	c.1570C>T	Non-sense	ENSG00000136531	no	-	-

Patient RNA expression level: The RNA expression quantity of indicated gene of the index ASD patient; Mean RNA expression level: The average value of RNA expression quantity of the indicated gene in total 354 ASD patients.

## Discussion

Large-scale genomic studies have revealed multiple CNVs and SNVs in the pathogenesis of ASD (Ruzzo et al., 2019; Satterstrom et al., 2020). In this study, we found 10 SNVs in 354 ASD children with a positive rate of 2.8%, and 9 CNVs with a positive rate of 2.5%. The total positive rate (5.3%) is relatively lower than previous studies (Sebat et al., 2007; Gaugler et al., 2014). The major reason is that we mostly focused on the known CNVs and *de novo* dominant protein-truncating SNVs. These mutations explain the genetic etiology of ASD with rare mutations. The pathological *de novo* mutations accounts for 4.2% (15/354) in our cohort. Other types of mutations, such as missense mutation of ASD genes, recessive SNVs, mosaicism, regulatory pathogenic variations or non-coding variants, may explain a large number of ASD in its genetic etiology (Zhang et al., 2021). The diagnostic rate of autism by other Chinese researchers ranges from 4% to 19% (Jiang et al., 2013; Wang et al., 2016; Du et al., 2018; Wu et al., 2018). The variable molecular diagnostic rate may be due to different cohorts of patients, detection methods and mutational types. It is possible in our pipeline that some pathogenic SNVs are missed due to the lower sequencing depth (∼30×) compared to regular trio-WES depth (usually > 100×). In addition, we did not exclude the patients with fragile X syndrome or those cases with inborn error of metabolism who present autistic symptoms by biochemical screening. It must be noted that we have uncovered dozens of candidate variants that are characterized as variants of uncertain significance (VUS) to be verified by functional assays as causative variants or genes for ASD.

Three patients with SNVs in our cohort were in the *SCN2A* gene, and two cases with CNVs were in the 15q11q13 recurrent region (PWS/AS, BP2-BP3, Class 2). This data suggests that these ASD genes are more common in Chinese ASD, which agrees with previous findings by other researchers (Wang et al., 2016; Guo et al., 2017).

The combination of WGS and CNVseq can improve the diagnostic rate to a certain extent. However, since the cause of most ASD is unclear, complex SV may be one of the genetic alterations. In subsequent studies, we will explore non-coding region variation and SV in relation to ASD to improve diagnostic rate, as well as to explore new ASD-causative genes in combination with functional assays and animal models.

The RNA sequencing results of peripheral blood of our positive patients were well consistent with expected changes, suggesting that peripheral blood RNAseq can also be used as a means of detection and verification for patients with autism. The gene expression level could be inferred from the expression in peripheral blood detection (Xu et al., 2020). However, some ASD genes are not expressed in peripheral blood, which will have certain limitations for the diagnosis of autism patients.

In our study, autistic patients with comorbidity are more likely to find the genetic etiology, especially those with intellectual disability and language retardation. This also suggests that WGS together with RNAseq is effective in identifying the cause of autism when it is accompanied by other comorbidities, which is important for early diagnosis and precise intervention of ASD.

## Data Availability

According to national legislation/guidelines, specifically the Administrative Regulations of the People’s Republic of China on Human Genetic Resources (http://www.gov.cn/zhengce/content/2019-06/10/content_5398829.htm, http://english.www.gov.cn/policies/latest_releases/2019/06/10/content_281476708945462.htm), no additional raw data is available at this time. The raw sequence data reported in this paper have been deposited in the Genome Sequence Archive (Genomics, Proteomics & Bioinformatics 2021, 19(4):578-583) in National Genomics Data Center, China National Center for Bioinformation/Beijing Institute of Genomics, Chinese Academy of Sciences (GSA-Human: HRA004176/Genetic Diagnostic Yields of 354 Chinese ASD Children with Rare Mutations by a Pipeline of Genomic Tests) that are publicly accessible at https://ngdc.cncb.ac.cn/gsa-human. Data of this project can be accessed after an approval application to the project leader, WL. Please refer to email: liwei@bch.com.cn for detailed application guidance. The accession number HRA004176 should be included in the application.
