# Supplementary material for: Genetic diagnostic yields of 354 Chinese ASD children with rare mutations by a pipeline of genomic tests
Source: Front Genet. 2023 Mar 23;14:1108440. doi: 10.3389/fgene.2023.1108440 (PMC10076746; doi:10.3389/fgene.2023.1108440)
Supplement: Supplementary file 1 [file DataSheet1.docx]

**Supplementary Materials**

1. **DSM-V for ASD diagnosis**

**A child must have persistent deficits in each of three areas of social communication and interaction (see A1 through A3 below) plus at least two of four types of restricted, repetitive behaviors (see B1 through B4 below).**

**A: Persistent deficits in social communication and social interaction across multiple contexts, as manifested by the following, currently or by history (examples are illustrative, not exhaustive; see text):**

A1: Deficits in social-emotional reciprocity, ranging, for example, from abnormal social approach and failure of normal back-and-forth conversation; to reduced sharing of interests, emotions, or affect; to failure to initiate or respond to social interactions.

A2: Deficits in nonverbal communicative behaviors used for social interaction, ranging, for example, from poorly integrated verbal and nonverbal communication; to abnormalities in eye contact and body language or deficits in understanding and use of gestures; to a total lack of facial expressions and nonverbal communication.

A3: Deficits in developing, maintaining, and understanding relationships, ranging, for example, from difficulties adjusting behavior to suit various social contexts; to difficulties in sharing imaginative play or in making friends; to absence of interest in peers.

**B: Restricted, repetitive patterns of behavior, interests, or activities, as manifested by at least two of the following, currently or by history (examples are illustrative, not exhaustive; see text):**

B1: Stereotyped or repetitive motor movements, use of objects, or speech (e.g., simple motor stereotypes, lining up toys or flipping objects, echolalia, idiosyncratic phrases).

B2: Insistence on sameness, inflexible adherence to routines, or ritualized patterns of verbal or nonverbal behavior (e.g., extreme distress at small changes, difficulties with transitions, rigid thinking patterns, greeting rituals, need to take same route or eat same food every day).

B3: Highly restricted, fixated interests that are abnormal in intensity or focus (e.g., strong attachment to or preoccupation with unusual objects, excessively circumscribed or perseverative interests).

B4: Hyper- or hypoactivity to sensory input or unusual interest in sensory aspects of the environment (e.g. apparent indifference to pain/temperature, adverse response to specific sounds or textures, excessive smelling or touching of objects, visual fascination with lights or movement).

**C: Symptoms must be present in the early developmental period (but may not become fully manifest until social demands exceed limited capacities, or may be masked by learned strategies in later life).**

**D: Symptoms cause clinically significant impairment in social, occupational, or other important areas of current functioning.**

**E: These disturbances are not better explained by intellectual disability (intellectual developmental disorder) or global developmental delay. Intellectual disability and autism spectrum disorder frequently co-occur; to make comorbid diagnoses of autism spectrum disorder and intellectual disability, social communication should be below that expected for general developmental level.**

1. **Supplementary Figures and Figure Legends**


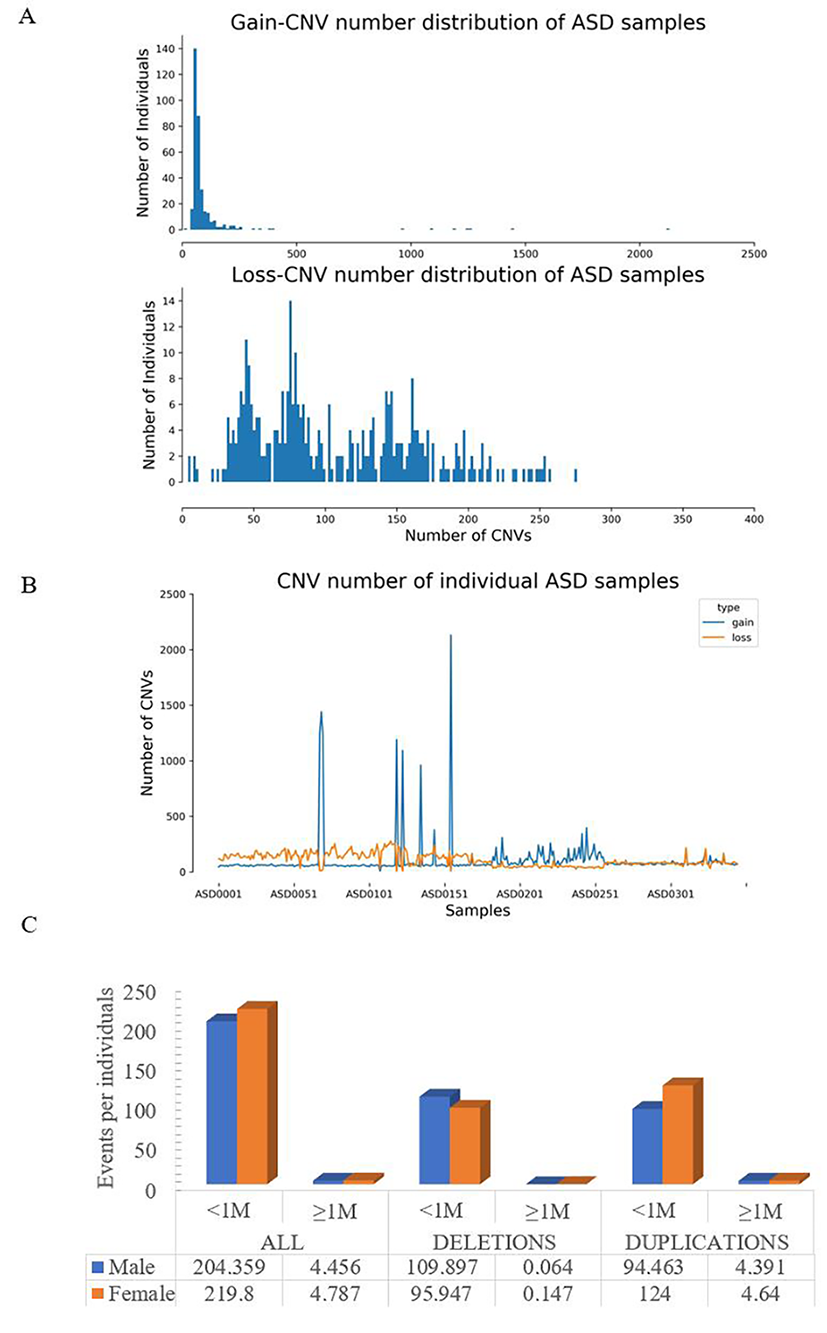


**Figure S1.** The distribution of CNV calls of individual cases. A. The distribution of gain-CNV and loss-CNV numbers of 354 patients. B. The distribution of CNV calls of each patient. C. Burden analysis of large CNVs in male and female patients. Deletions, duplications and the combined rate for all CNVs are shown. The CNV size was categorized as < 1 Mb and ≥1 Mb.

.

**
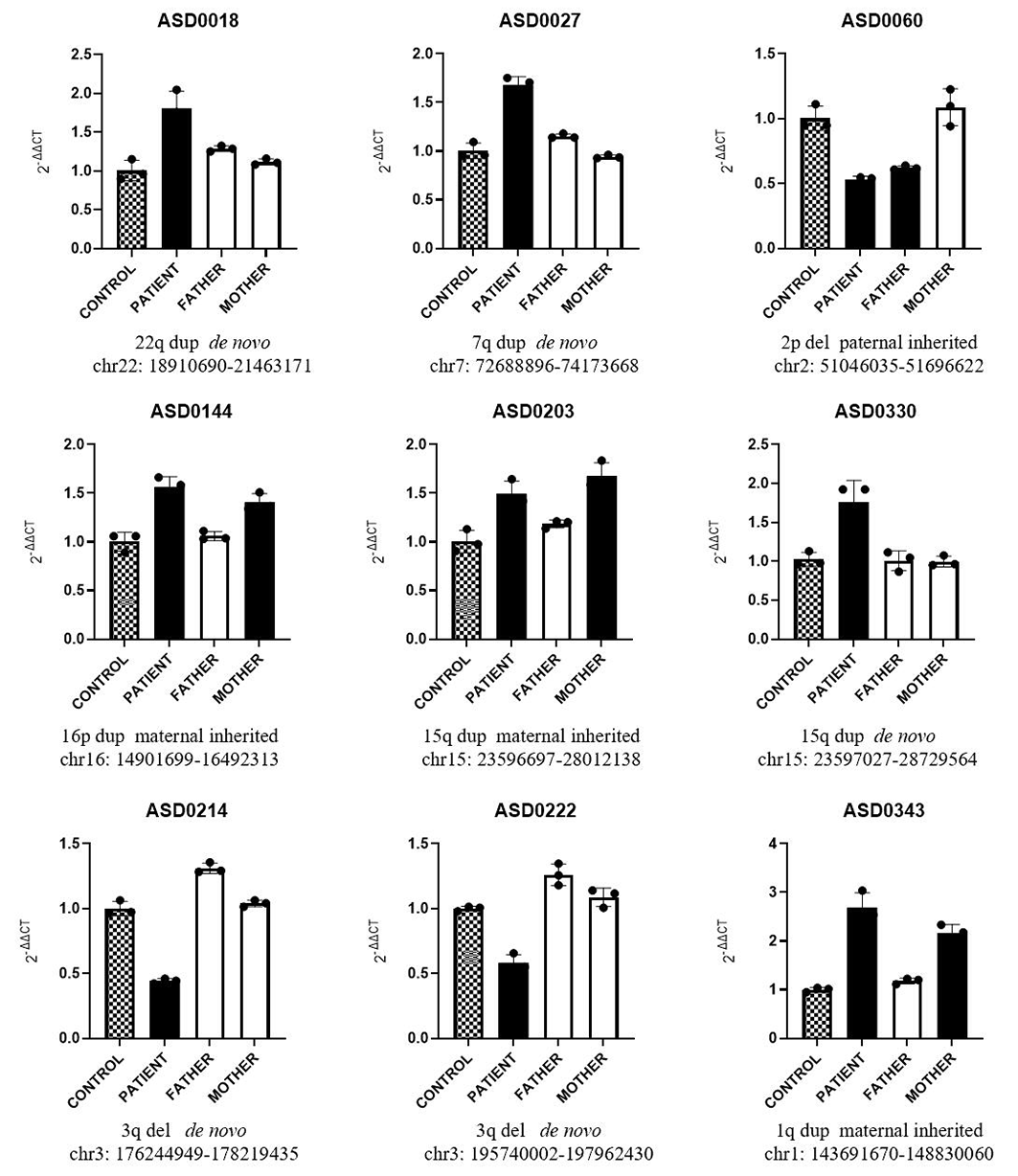
**

**Figure S2**. qPCR validation of CNVs detected in 9 ASD families. Dotted bars represent the normal control, which were set to a value of 1. Black bars represent the patients and their affected parents who carried the same copy number variations. White bars represent the unaffected parents. In the 9 ASD families, there were 5 *de novo* CNVs and 4 inherited CNVs.


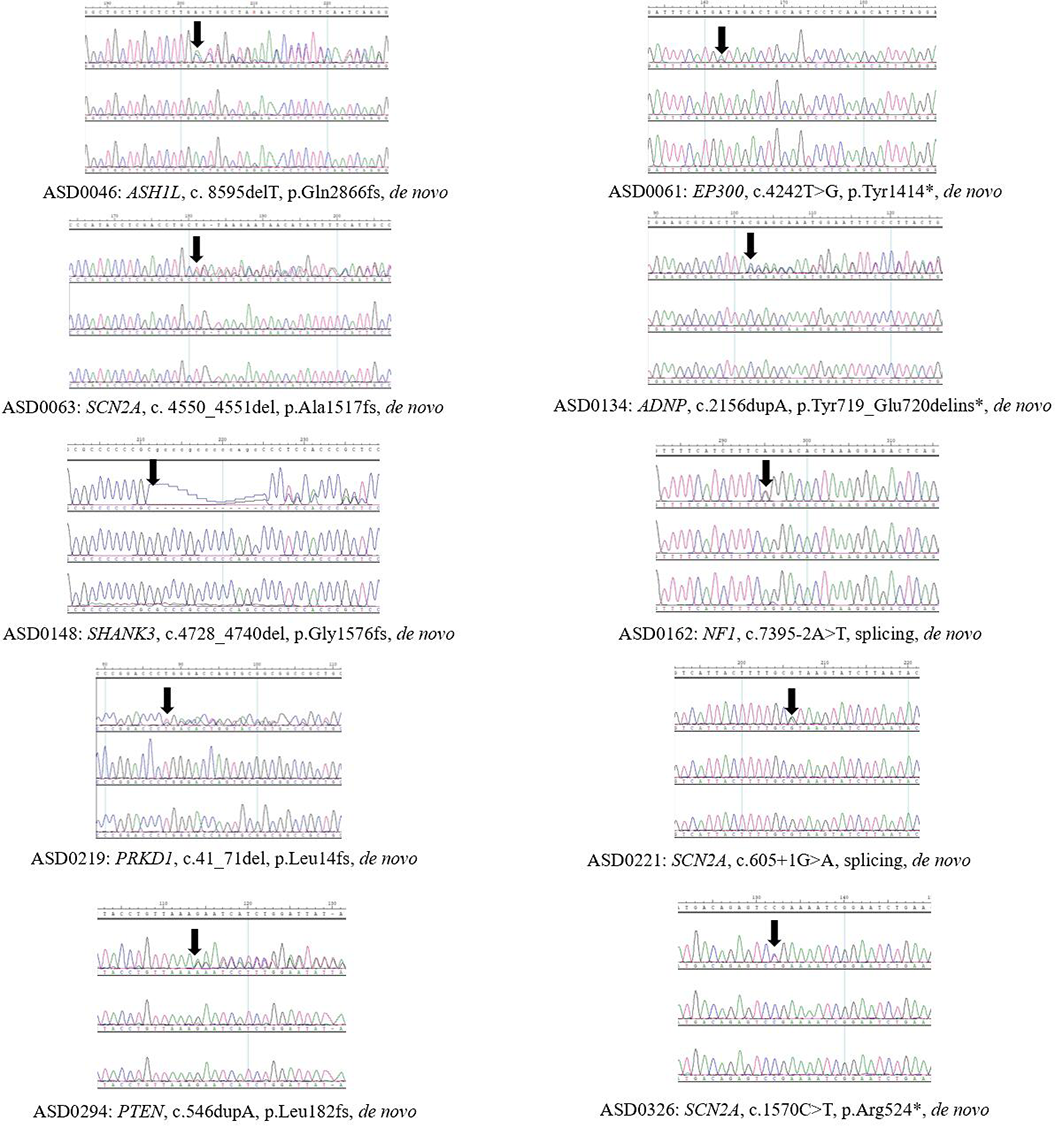


**Figure S3.** Validation of *de novo* pathogenic SNVs in 10 ASD families. The mutation sites are indicated by the black arrows in the histograms of Sanger sequencing.
